# The Role of Moral Suffering (Moral Distress and Moral Injury) in Police Compassion Fatigue and PTSD: An Unexplored Topic

**DOI:** 10.3389/fpsyg.2017.01999

**Published:** 2017-11-15

**Authors:** Konstantinos Papazoglou, Brian Chopko

**Affiliations:** ^1^Department of Psychology, University of Toronto, Toronto, ON, Canada; ^2^Department of Sociology, Kent State University, North Canton, OH, United States

**Keywords:** police trauma, PTSD, resilience, moral distress, moral injury, compassion fatigue, resilience promotion, health promotion

## Traumatization and moral suffering among police officers

In the early spring of 2014, the first author was collecting data through a field research study that was conducted during a police special forces tactical training session. In one of the critical incident training scenarios (a hostage situation), the person playing the role of the violent criminal was lying on the ground pretending to be severely injured as a result of being shot by the officers. When the incident had been resolved and the hostages were safe, one of the police officers began to administer first aid care to both the criminal and the wounded hostages. When asked why he chose to treat the criminal as well as the victims, he responded that, “We are cops, we are not killers. We need to take care of everybody injured in the scene.” The author contends that such scenarios (e.g., the attempt to take care of a criminal who tried to kill you, your fellow officers, or civilians) generate moments of moral suffering. Moral suffering's prominent role toward traumatization has been highlighted by many trauma scholars (e.g., Litz et al., 2009) who suggest that current trauma research has not efficiently investigated the phenomenon of moral suffering in frontline professionals' exposure to traumatic incidents (Nash and Litz, 2013; Kopacz et al., 2016; Maguen and Litz, 2016). In addition, it is argued that current post-traumatic stress disorder (PTSD) diagnostic criteria do not efficiently capture the phenomenon of moral suffering in frontline professionals' exposure to traumatic incidents (Nash and Litz, 2013).

In his seminal book “*Treating compassion fatigue*,” Vietnam veteran and trauma research professor Figley (2002) mentioned that compassion fatigue refers to a type of secondary traumatic stress that emanates from frontline professionals' “cost of caring” for those who suffer psychological pain. Further, Figley (2002) noted that compassion fatigue is related to professional caregivers' cognitive schemata; some of those cognitive schemata are akin to frontline professionals' morale. Based on Figley's (2002) perspective, it seems that compassion fatigue is cognitively related to moral suffering experienced by first responders in the line of duty. Scholarly work after Figley's (2002) earlier work has specified and further examined two types of moral suffering that appear to lead to traumatization; that is, moral distress and moral injury. It is appeared from the literature that moral distress and moral injury are distinct and emanate from different morally conflicted incidents. However, both moral distress and moral injury may lead to police traumatization. In the following paragraphs, authors discuss moral distress and moral injury and their relationship to traumatization. The role of moral distress and moral injury toward traumatization (compassion fatigue and PTSD) has never been studied in police empirically. This present manuscript is the first in this area and aims to theoretically conceptualize the role of moral suffering (moral distress and moral injury) toward police traumatization (compassion fatigue and PTSD). Due to lack of empirical research with police in this area, the authors reviewed and discuss in this manuscript research studies that conducted with frontline professionals (e.g., therapists, social workers, nurses) as well as soldiers and veterans. That way, the authors posited that previous research findings with frontline professionals and veterans/soldiers in this area may be utilized to open theoretical dialogues that will help us examine the role of moral suffering toward traumatization in police.

## Moral distress and police work

Moral distress was first talked about by nursing professionals and scholars who emphasized the challenging moral moments that nurses experience when they provide care to patients and their families (Elpern et al., 2005). In the mid-80s, philosopher Jameton (1984) defined moral distress as the experience of painful feelings and psychological disequilibrium that takes place when nurses are aware of a morally proper decision that needs to be made without being able to make it, usually as a result, of various hurdles: institution policy, lack of time, protocol, and so forth. Other scholars have suggested that moral distress refers to situations when caregiving professionals fail to pursue the right course of action because of an error in judgment, wrong decision-making, wrong action plan, or the fact that the circumstances are beyond their action planning (Kälvermark et al., 2004). Further, McCarthy and Deady (2008) argued that moral distress is pervasive among caregiving professionals who are expected to make decisions and judgments—that encompass moral components—during complex incidents. To date, caregiving professionals such as occupational therapists (Penny et al., 2016) and social workers (Mänttäri-van der Kuip, 2016) have emphasized the incapacitating role of moral distress on caregiving professionals' health, job performance, and wellbeing. As noted, moral distress has been studied in nurses, therapists, and social workers. Authors intend to emphasize on the fact that police officers may also experience moral distress in the line of duty and raise awareness among researchers and clinicians that moral distress in police has not been empirically studied yet.

In this way, police are mandated to maintain peace and order, provide compassion to victims of crimes and accidents, and to save those who are in danger. From the time they first join the police academy, police officers are instilled with the prominent role dedication, integrity, and even self-sacrifice ought to play in their conduct, with the objective in mind to save and support civilian victims and to complete their missions successfully. In many occasions, the “God's syndrome” (Beaton and Murphy, 1995, p. 69) is pervasive among police officers which refers to officers' attempt to respond to all emergency calls, save all victims, support all those who suffer. Nevertheless, according to the circumstances they find themselves in, first responders are not always able to protect or support victims, or arrest violent criminals. However, when police officers' action plans, or willingness to help those who suffer is precluded or is not completed successfully, then officers may experience moral distress (Corley, 2002; Morley, 2003).

## Moral distress and police traumatization

The ongoing experience of moral distress may lead to compassion fatigue, which may eventually lead officers to experience PTSD as well as other comorbid disorders (e.g., major depressive disorder, panic disorder) (Andersen and Papazoglou, 2015). In an article in the Royal Canadian Mounted Police (RCMP) magazine (so-called “Gazette”), police clinical psychologist, Morley (2003) stated that moral distress refers to an on-going “unfixable suffering” that may accumulate over time in service and eventually lead to compassion fatigue. Likewise, researchers have argued that moral distress among caregiving professionals may eventually lead to compassion fatigue, emotional paralysis, avoidance of response to certain critical incidents, and even job resignation (Beaton and Murphy, 1995; Sundin-Huard and Fahy, 1999). In addition, moral distress may lead caregiving professionals to helplnessness, powerlessness, shame, embarrassment, compromised integrity and justice, reduced sense of dignity, grief, misery, and anguish (Corley, 2002; Elpern et al., 2005); yet, the role of moral distress in compassion fatigue as well as PTSD symptomatology and treatment has not been studied empirically. The empirical examination of the role of moral distress toward traumatization may enlighten our understanding to pathways that lead toward police traumatization. In turn, such findings may help us develop preventative interventions that may promote resilience among police.

## Moral injury and police work

Moral injury has been predominantly developed from clinical work and research with soldiers and veterans. Moral injury refers to unprecedented traumatic life events that refer to perpetrating, failing to prevent, or bearing witness to actions that “transgress deeply held moral beliefs and expectations” (Litz et al., 2009, p. 1). Morally injured individuals, for instance, often alter their beliefs that the world is a safe and benevolent place and human beings trustworthy. Events that may lead to moral injury may be death-related situations, killings, handling/uncovering human remains, severely wounded victims that the person was not able to help (Frankfurt and Frazier, 2016). Similar to moral distress, individuals who suffer from moral injury may experience guilt, frustration, sense of rejection, difficulty forgiving, self-harm, anhedonia, and shame (Nash and Litz, 2013; Shay, 2014). It is argued that moral injury is one of the precursors of PTSD symptoms (Nash and Litz, 2013). As well, Shay (2014) contended that moral injury is prevalent in a number of PTSD clustered-symptoms, specifically: the nature of triggering an event, re-experiencing, and avoidance or numbing.

Presently, recent research has initiated to empirically study moral injury experienced in soldiers and veterans. However, current literature has not addressed (empirically or even theoretically) moral injury experienced by police. Similarly to authors' perspective on moral distress, present manuscript aims to make researchers and clinicians aware that is imperative that moral injury in police should be studied empirically. Nonetheless, police officers often experience atrocities, gruesome crimes, and death in the line of duty (Papazoglou, 2013).

## Moral injury and police traumatization

Analogously, to the experience of veterans and soldiers, police officers are expected to experience moral injury that may be a precursor to compassion fatigue and, intertwined with compassion fatigue, a risk factor to PTSD susceptibility and severity. To illustrate, approximately 25% of a police sample reported killing or seriously injuring a suspect in the line of duty (Weiss et al., 2010). Of 60 different operational and organizational stressors common to the policing profession, officers ranked killing someone during a use of force encounter as the most stressful experience in terms of coping with the aftermath (Violanti and Aron, 1995). Interestingly, officers conveyed in two different studies that making a mistake in the line of duty resulting in the death of a colleague would be the most stressful (also in terms of coping with the aftermath) from a list of 34 potentially traumatic experiences (e.g., being threatened with a gun, witnessing someone being killed) commonly experienced by officers (Weiss et al., 2010; Chopko et al., 2015).

Thus, underlying feelings of guilt and shame (i.e., moral injury) and resulting distress related to the harming of others experienced by law enforcement may have commonalities with soldiers. Komarovskaya et al. (2011) found that killing or severely injuring a perpetrator was significantly related with PTSD symptoms among police officers. Noteworthy, active-duty law enforcement officers reported experiencing higher levels of PTSD symptoms compared to active-duty U.S. infantry soldiers soon after returning from combat missions in Iraq and Afghanistan (Chopko et al., under review). Furthermore, subjective ratings of overall posttraumatic stress were more strongly related to maladaptive outcomes (e.g., dysfunctional alcohol use) compared to PTSD symptoms among police officers (Chopko et al., 2013). The authors of this research postulated that guilt and shame related to traumatic experiences might have accounted, at least partially, for this finding. Lastly, Chopko et al. (2016) reported that greater posttraumatic spiritual growth was positively related with greater distress (e.g., PTSD, depression) among overwhelmingly Christian (i.e., “Thou shall not kill”) officers. The authors of this research proposed that a spiritual quest is initiated by traumatic experiences to cope with guilt and shame surrounding those events (e.g., harming a fellow human being).

## Moral distress vs. moral injury

Moral distress and moral injury may appear to be very similar since both are related to moral suffering. However, what appears to mainly distinguish moral injury from moral distress is that moral injury refers to violence and death-related incidents. On the other hand, moral distress refers to moral dilemmas (or else moral disequilibrium) experienced by multiple mundane incidents that frontline professionals may frequently experience in the line of duty. To this end, moral injury, for instance, may be experienced with intense sense of feelings of guilt, shame, and frustration that an officer may experience because of exposure to extreme violence or death-related incidents. On the other hand, officers may experience multiple incidents that may cause moral distress; over time, guilt, shame, and other feelings may accumulate due to multiple morally distressed causing incidents in the line of duty.

From a theoretical perspective it may be inferred that moral distress and moral injury are intertwined in police work and may lead officers to the experience of compassion fatigue. In turn, the continuous experience of multiple critical incidents that lead to the experience of moral distress, moral injury, and compassion fatigue, may render officers more vulnerable to the experience of PTSD symptomatology. That is, it is posited that the officers' experiences of both moral distress and moral injury are cumulatively intertwined and eventually may lead toward traumatization (compassion fatigue, PTSD).

## Conclusions and future research

The issue of moral suffering (moral distress and moral injury) should be also studied within a law-enforcement context given that all police officers are likely to experience moral suffering in the line of duty. Such incidents may take multiple forms during an officers' service, including: officer vs. officer, officer vs. civilian, and officer vs. organization. Although officers often explore their options so that they act in a way that is consistent with their own morals and beliefs, they may nevertheless experience inner moral suffering on multiple occasions throughout a shift. For example, an officer may experience inner moral suffering if his partner acts against a civilian in a manner that he considers morally wrong, or he may need to act in a way that is at odds with his moral values in order to comply with the organization's policies (or their supervisor's orders). One such instance where conflict may arise between an officer's personal moral beliefs and their duty to adhere to departmental policy or a direct order is crowd management. In crowd-control situations, officers may be ordered to use force on protestors—some of whom may be teenagers—and such action may be inconsistent with an officer's personal moral beliefs. Similarly, an officer may view her partner's behavior toward an elder as disrespectful, but she may elect to not say anything in order to avoid conflict with her partner. The authors contend that such inner moral suffering become intensified when officers are exposed to critical incidents, which are defined in the literature as the moments in which morally injurious incidents occur (e.g., Litz et al., 2009). Such incidents may not be traumatic *per se*, but they can be potentially morally injurious because they may lead first responders to question their tactical decision-making in response to the incident, their capacity to prevent what happened, and so forth. For instance, officers are called to respond to a domestic violence situation, but their arrival is delayed due to traffic congestion. When they finally arrive, they find two family members severely wounded. In this scenario, the officers may question whether there was anything they could have done differently to prevent that incident from happening. In addition, their actions may be reviewed by their shift supervisor to determine whether they had performed their duties as they were supposed to. Such an incident may not lead officers toward PTSD or compassion fatigue *per se*. However, it is possible that the officers in this scenario may have experienced moral suffering (especially if they were found accountable by their shift supervisor) due to feelings of guilt and shame about the incident's outcome. Morally injurious incidents are omnipresent in police work; thus, incorporating moral suffering (moral distress and moral injury) into the present study is of great value because it represents a possible “entrance gate” that can allow for the examination of the different pathways that officers follow and that make them susceptible to traumatization. Of course, police work is not solely caring for and protecting those who suffer. Many times, police officers are supposed to apply use of force or even lethal use of force, if needed. In some occasions, police brutality, excessive use of force, unnecessary use of lethal force, and negative behavior toward civilians may occur. It is possible that some (not all) of those cases may happen because police officers are likely to experience moral suffering and/or traumatization that precludes their ability to perform their duties, make the right decisions, or even regulate their negative emotions (e.g., anger, frustration) efficiently. Nevertheless, future research is imperative to shed light to such cases where officers' show negativity, hostility, or unnecessary use of force to civilians.

Even though it was out of the main scope of this manuscript, authors would like to emphasize that literature has also been abandoned on the impact of police stress work on officers' wellbeing (e.g., Rothmann, 2008; Tomyn et al., 2015). Specifically, officers' negative coping strategies (e.g., avoidance, self-criticism) (Knowles and Bull, 2003) and lack of work-related effective stress management (Mostert and Rothmann, 2006) essentially decreased their wellbeing. On the other hand, balanced workload, supportive relationships by peers and supervisors, and sense of self-control appear to reverse the egregious impact of work-related stress on officers' wellbeing (Tomyn et al., 2015; Hesketh et al., 2016). Those literature findings may support future research and policy programs in trauma prevention as well as wellbeing promotion among police officers.

To this end, the authors concisely suggest the following action plan for future research, policy, and clinical work that should be considered in working with police officers:

- Empirical research aims to study the role of moral distress and moral injury in the experience of compassion fatigue among police officers.
- Development of standardized psychosocial scales intended to assess moral distress and moral injury among first responders.
- Empirical research aims to study the role of moral distress and moral injury in officers' susceptibility to PTSD, as well as in the severity of their PTSD symptoms.
- Development of evidenced-based PTSD treatment that addresses issues such as moral injury and moral distress.
- Police academies in collaboration with health care professionals to offer training for prevention of moral distress and moral injury, and promotion of moral resilience.

## Author contributions

Both authors conducted thorough review of the literature. KP developed the first half of the opinion article that refers to the theoretical conceptualization of the topic. In addition, first author wrote up paragraphs relative to challenges of police work in the line of duty. BC worked on paragraphs that refer to the role of guilty and trauma in police work. The idea of the article was conceptualized by the KP. However, both authors worked closely for the final version of this article that is now submitted to the Frontiers in Psychology

### Conflict of interest statement

The authors declare that the research was conducted in the absence of any commercial or financial relationships that could be construed as a potential conflict of interest.
